# Cocaine and Its Use in Ophthalmic and General Surgery

**Published:** 1885-05

**Authors:** 


**Cocaine and its use in Ophthalmic and General Surgery.** By H. KNAPP, M. D., Professor of Ophthalmology in the Medical Department of the City of New York. Reprinted from the "Archives of Ophthalmology," December, 1884, with Supplementary Contributions by Drs. F. H. BOSWORTH, R. J. HALL, E. L. KEYES, H. KNAPP and W. H. POLK. New York and London: G. P. Putnam's Sons, The Knickerbocker Press. 1885.

This monograph on cocaine contains the latest opinion and experiences concerning this most useful remedy. The author's well-known ability and wide reputation in ophthalmology is an ample guarantee that he presents the latest experience of accurate observers as to the application of this remedy in ophthalmic and surgical practice. He has called to his aid several able collaborators, who furnish valuable articles on its application to other departments of medicine. Dr. Bosworth gives a chapter on cocaine in the upper air passages; Dr. Hill on general surgery; Dr. Keyes on genito-urinary and minor surgery, and Dr. Polk on gynæcology and obstetrics. It will be seen that the accomplished author has succeeded in presenting a work of rare value to the profession.

